# Energy Efficient Artificial Olfactory System with Integrated Sensing and Computing Capabilities for Food Spoilage Detection

**DOI:** 10.1002/advs.202302506

**Published:** 2023-08-31

**Authors:** Gyuweon Jung, Jaehyeon Kim, Seongbin Hong, Hunhee Shin, Yujeong Jeong, Wonjun Shin, Dongseok Kwon, Woo Young Choi, Jong‐Ho Lee

**Affiliations:** ^1^ Department of Electrical and Computer Engineering and Inter‐University Semiconductor Research Center Seoul National University Seoul 08826 Republic of Korea; ^2^ Ministry of Science and ICT Sejong 30121 Republic of Korea

**Keywords:** artificial olfactory system, electronic nose, FET‐type gas sensor array, food spoilage detection, near‐sensor computing, nonvolatile memory (NVM) array

## Abstract

Artificial olfactory systems (AOSs) that mimic biological olfactory systems are of great interest. However, most existing AOSs suffer from high energy consumption levels and latency issues due to data conversion and transmission. In this work, an energy‐ and area‐efficient AOS based on near‐sensor computing is proposed. The AOS efficiently integrates an array of sensing units (merged field effect transistor (FET)‐type gas sensors and amplifier circuits) and an AND‐type nonvolatile memory (NVM) array. The signals of the sensing units are directly connected to the NVM array and are computed in memory, and the meaningful linear combinations of signals are output as bit line currents. The AOS is designed to detect food spoilage by employing thin zinc oxide films as gas‐sensing materials, and it exhibits low detection limits for H_2_S and NH_3_ gases (0.01 ppm), which are high‐protein food spoilage markers. As a proof of concept, monitoring the entire spoilage process of chicken tenderloin is demonstrated. The system can continuously track freshness scores and food conditions throughout the spoilage process. The proposed AOS platform is applicable to various applications due to its ability to change the sensing temperature and programmable NVM cells.

## Graphical abstract

A novel near‐sensor computing‐based artificial olfactory system (AOS) that can be implemented to perform energy‐ and area‐efficient sensing and computing functions on the same chip is presented. AOS is applied to food spoilage detection and AOS successfully provides real‐time food conditions continuously throughout the chicken tenderloin spoilage process.

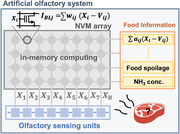

## 1 Introduction

Biological olfactory systems (BOSs) play essential roles in living things, such as predator detection and food freshness determination.^[^ ^1^, ^2^, ^3^ ^]^ Since the concept of an electronic nose (EN) that imitates BOSs was presented in 1982,^[^ ^4^ ^]^ research on artificial olfactory systems (AOSs) has steadily increased.^[^ ^5^, ^6^, ^7^, ^8^, ^9^, ^10^, ^11^, ^12^, ^13^ ^]^ AOSs have been commercialized and utilized in numerous fields, including disease monitoring and food spoilage detection.^[^ ^6^, ^7^ ^]^ Owing to the development of complementary metal–oxide–semiconductor (CMOS)‐compatible gas sensors, such as GasFET,^[^ ^14^, ^15^ ^]^ capacitively coupled FET (CCFET),^[^ ^16^, ^17^ ^]^ and floating‐gate FET (FGFET),^[^ ^18^, ^19^ ^]^ AOSs integrated with a large‐scale gas sensor array with digital‐based interface circuits have been implemented.^[^ ^20^, ^21^, ^22^ ^]^ The performance of AOS has been improved by applying a machine learning‐based gas identification algorithm using a large amount of gas sensor data.^[^ ^23^ ^]^

Conventional AOSs consist of sensor arrays, analog‐to‐digital converters (ADCs), microcontroller units (MCUs), memories, processors, and servers.^[^ ^7^, ^8^, ^9^, ^24^, ^25^ ^]^ Olfactory information is typically generated by converting analog sensor array signals into digital signals and by passing them to local processors or servers for processing and inference. Since the sensor devices and computing devices are physically separated and data processing and inference are performed in a centralized von Neumann computing architecture, conventional AOSs have certain limitations, such as high energy consumption, latency, and data loss.^[^ ^26^, ^27^, ^28^ ^]^ These limitations intensify as larger AOSs with more sensors are developed. In addition, to implement portable AOS in a variety of edge devices, these limitations must be addressed.

Recently, energy‐efficient architectures that perform sensing and computation functions on the same chip have received increasing research interest.^[^ ^27^, ^28^, ^29^, ^30^, ^31^, ^32^ ^]^ Energy‐efficient AOSs have been proposed using 3D integrations,^[^ ^22^, ^33^ ^]^ memristors,^[^ ^34^, ^35^ ^]^ and spiking neurons.^[^ ^36^, ^37^ ^]^ Vertically stacking processors, memories, and sensor array layers on a single chip are favorable for the size and communication speed; however, fabrication is challenging, and digital signal‐based sensor/processor interfaces are still required.^[^ ^22^, ^33^ ^]^ However, devices that employ in‐memory computing (IMC) using memristors and signal processing through spiking neurons have simple interfaces, and they effectively reduce energy consumption. Nevertheless, to acquire reliable olfactory information utilizing IMC and spiking neurons, the raw data from sensors should undergo low‐level processing, such as baseline cancellation and noise reduction.^[^ ^28^ ^]^

Among various olfactory sensors, semiconducting metal oxide (SMO) sensors have received the most attention due to their ability to be fabricated on a large scale using CMOS‐compatible processes.^[^ ^38^ ^]^ SMO‐based sensors have been successfully commercialized because of their high sensitivity and reliability. However, SMO‐based sensors require high operating temperatures (>100 °C). There have been proposals for sensors operating at room temperature (RT);^[^ ^39^, ^40^, ^41^ ^]^ however, there are still concerns with the humidity effect, long recovery time, device‐to‐device variation, and mass production. For example, electrochemical and polymer‐based gas sensors have shown excellent performance at room temperature and are being developed to overcome these concerns.^[^ ^42^, ^43^ ^]^ In SMO‐based sensors, microheaters are built into sensors, and the heaters consume a large amount of energy.^[^ ^44^, ^45^ ^]^ Therefore, to apply AOSs to edge devices, purpose‐oriented AOSs must be designed based on the small number of sensors and the optimal heater operation (e.g., pulsed operation).

**Figure 1:**
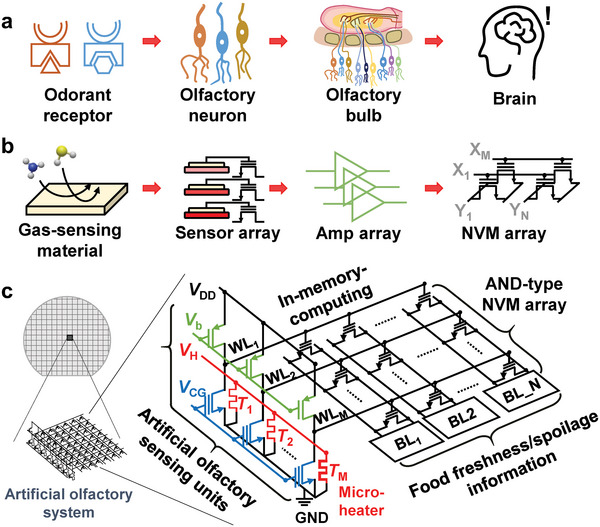
Biological olfactory system and proposed artificial olfactory system. a) Sequence of odor detection by the biological olfactory system. b) Sequence of gas detection by the proposed artificial olfactory system. c) Schematic diagram of the proposed artificial olfactory system.

In this work, we present a near‐sensor computing‐based AOS for food spoilage detection. The proposed AOS has a high energy efficiency and compact design by mimicking the BOS. The BOS obtains olfactory information in the following manner (**Figure** **1**a). As odorant receptors react with volatile molecules and produce chemicals, olfactory neurons generate electrical signals that are transmitted to the olfactory bulb.^[^ ^46^ ^]^ The olfactory data pattern of the olfactory bulb is transmitted to the olfactory cortex of the brain, and olfactory information is inferred.^[^ ^47^ ^]^ In the proposed AOS, a sensing operation is performed through a gas sensing material, a gas sensor array containing the sensing material, an amplifier array containing gas sensors, and an NVM array (Figure 1b). The charge transfer caused by the adsorbate‐surface reaction in the sensing material changes the electrical properties of the gas sensor. The proposed AOS consists of an amplifier array and an AND‐type NVM array integrated on the same substrate using conventional CMOS process technology (Figure 1c). In the amplifier array, each amplifier consists of a FET‐type sensor and a load FET to reduce area and power consumption. The sensed signal is amplified and converted to a voltage by an amplifier before being passed to the WL in a non‐volatile memory (NVM) array. In the NVM array, olfactory sensing data are linearly combined through IMC‐based multiplication and accumulation (MAC) operations, and meaningful olfactory information is provided as output. The proposed AOS uses a novel method of integrating the output of the sensing unit and the input of the in‐memory computing block without using the sensor/processor interface circuits and conventional low‐level processing. To verify the performance of the proposed AOS in real‐world applications, we demonstrate that AOS can provide continuous food (chicken tenderloin) spoilage information as an example.

## 2 Results and Discussion

### 2.1 Structure of the Artificial Olfactory System

**Figure 2:**
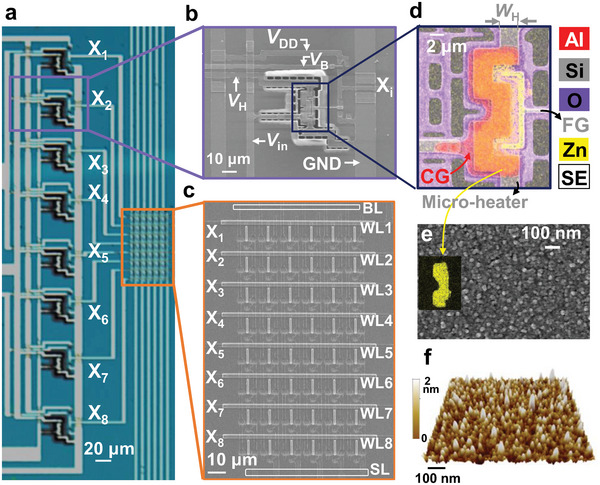
Design of the fabricated artificial olfactory system. a) Optical micrograph of the artificial olfactory system. b) SEM images of the artificial olfactory system unit and c) AND‐type NVM array. d) Energy‐dispersive X‐ray spectroscopy (EDS) mapping image of the fabricated gas sensor. e) Top SEM image of the ZnO film. f) AFM image of the ZnO film.

The proposed AOS is fabricated using standard CMOS technology (**Figure** **2**a; Figure S1, Supporting Information). The AOS integrates sensing units (Figure 2b; Figure S2, Supporting Information) and computing units (NVM array, Figure 2c). The fabricated AOS has eight sensing units, and the size of the fabricated NVM array is eight‐word lines (WLs) × six‐bit lines (BLs). The sensing unit consists of a gas‐sensing material, an *n*FET sensor, and a *p*FET load. The sensors feature control gates (CGs) and floating gates (FGs) in their constructions that are placed horizontally; low‐power microheaters are embedded beneath the CGs (Figure 2d).^[^ ^48^ ^]^ Gas sensors using floating gates have been studied for over 20 years and have shown excellent performance.^[^ ^20^, ^49^ ^]^ As a gas‐sensing material, an 8‐nm‐thick ZnO film is formed on the CG, and a SiO_2_/Si_3_N_4_/SiO_2_ (O/N/O) layer covers the FG (Figure 2d–f). The sensors have *n* ^+^ poly‐Si microheaters of varying widths (*W*_H_). When the heater voltage (*V*_H_) is supplied, the eight sensors operate at various temperatures (*T*_1_–*T*_8_) and exhibit different gas responses. An *n*FET sensor is connected in series with a *p*FET load to form a trans‐impedance amplifier circuit. The outputs of the sensing units (*V*_out_s = X_1_∼X_8_) are connected to the WLs of the AND‐type NVM array.

The proposed AOS directly uses the outputs of the sensing units as the inputs of the computing units without low‐level processing or interfaces. Unlike conventional AOSs, the proposed AOS does not require baseline compensation and noise reduction. The sensing units of conventional AOSs utilize gas‐sensing materials as resistors with varying conductance in response to the gas reaction.^[^ ^50^, ^51^ ^]^ As the current flows through the polycrystalline sensing material, the baseline of the signal drifts,^[^ ^52^ ^]^ and the sensing signals are very noisy.^[^ ^53^ ^]^ However, our gas sensors with Si FET transducers exhibit little baseline drift because the current does not flow through the gas‐sensing material (Figure S3, Supporting Information). In addition, since our sensors use crystalline Si as channels, our sensors can have ∼10^5^ times lower low‐frequency noise than conventional resistor‐type sensors.^[^ ^54^, ^55^ ^]^ Table S1 (Supporting Information) shows the performance comparison with state‐of‐the‐art AOSs with sensing and computing capabilities. Unlike previous studies, the proposed AOS uses analog signals to perform sensing and processing on the same chip, making it energy efficient.

### 2.2 Characteristics of the Artificial Olfactory Sensing Units

**Figure 3:**
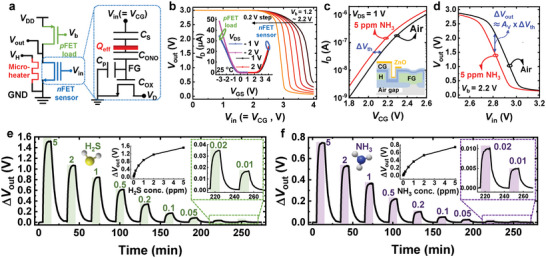
Characteristics of the artificial olfactory sensing unit. a) Schematic circuit diagram of the artificial olfactory sensing unit. *Q*_eff_, *C_S_*, *C*_ONO_, *C*_p_, and *C*_ox_ are the effective charge generated by the gas reaction, the capacitance of the sensing material, the capacitance of the O/N/O passivation layer, parasitic capacitance, and gate oxide capacitance, respectively. b) Transfer curves of the artificial olfactory sensing unit (amplifier circuit) as a parameter of gate bias (*V*_b_) of load *p*FET. Transfer curves of the *n*FET sensor and *p*FET load are shown in the inset. c) Transfer curves of the sensor and d) amplifier circuit before and after the 5 ppm NH_3_ gas reaction. A schematic cross‐section of the sensor is shown in the inset of (c). e) Dynamic responses of the artificial olfactory sensing unit to varying concentrations of H_2_S and f) NH_3_ gases at 265 and 222 °C, respectively. In the colored and uncolored regions, the sensing unit was exposed to gas and air, respectively. Δ*V*_out_ versus gas concentration curves are shown in the insets.

The equivalent circuit diagram and transfer curves of the artificial olfactory sensing unit are shown in **Figure** **3**a,b, respectively. Unlike existing sensing units that integrate each manufactured sensor and amplifier,^[^ ^7^, ^8^, ^33^ ^]^ the proposed sensing unit efficiently merges a sensor and an amplifier. The sensing unit detects gas through the following process. The interaction between the gas‐sensing material and gas produces an effective charge (*Q*_eff_). This *Q*_eff_ changes the threshold voltage (*V*_th_) of the sensor,^[^ ^48^ ^]^ which in turn changes the *V*_out_ of the sensing unit. That is, the sensing unit detects the adsorbate–surface reaction, amplifies the signal, and provides a voltage as an output. Figure 3c shows the transfer curves of the sensor before and after the gas reaction to 5 ppm NH_3_. Since NH_3_ is a reducing gas that reacts with pre‐adsorbed oxygen,^[^ ^56^ ^]^ the gas reaction forms a positive *Q*_eff_ and reduces the *V*_th_ of the sensor. Δ*V*_th_ causes a decrease in *V*_out_ of the amplifier (Figure 3d). Due to the complement of the *n*FET sensor and *p*FET load, the gain of the amplifier (*A*_V_ = Δ*V*_out_/Δ*V*_in_ ≈ Δ*V*_out_/Δ*V*_th_) is large (*A*_V_ = 9.3 V/V), maximizing the output signal (Figure S4, Supporting Information). The voltage (*V*_b_) applied to the gate of the *p*FET load is set to a value that maximizes *A*_V_ (Figure 3b).

The sensing units are optimized for high‐protein food spoilage detection. The conditions of high‐protein foods, such as meat, can be classified as fresh, edible, spoiled, or completely spoiled depending on the presence of gas, including no gas, traces of NH_3_ gas, large amounts of NH_3_ gas, and H_2_S gas.^[^ ^8^, ^57^ ^]^ ZnO is sensitive to NH_3_ and H_2_S gases, and it exhibits substantially different reaction characteristics to these gases.^[^ ^34^, ^58^, ^59^ ^]^ The X‐ray photoelectron spectroscopy (XPS) and grazing incidence X‐ray diffraction (GIXRD) analyses of the ZnO films are shown in Figures S5 and S6 (Supporting Information), respectively. Thin‐film type ZnO enables high process uniformity and gas sensitivity. The thin ZnO film increases the depletion capacitance (*C*_S,_ in Figure 3a) of the sensing material due to oxygen adsorption, thus increasing the effects of *Q*_eff_, Δ*V*_th_, and Δ*V*_out_.^[^ ^48^ ^]^ The responses of the sensing unit to H_2_S and NH_3_ gases at varying concentrations are shown in Figure 3e,f, respectively. Due to the low noise and high *A*_V_ of the sensing unit, the sensing unit has very low detection limits (DLs) (<0.01 ppm NH_3_ and 0.01 ppm H_2_S). The DLs of our sensing units are either similar to or surpass those of previously reported high‐performance sensors.^[^ ^8^, ^34^, ^60^ ^]^ Notably, the proposed sensing unit utilizes a film‐type gas‐sensing material that is highly reliable and advantageous for mass production.

**Figure 4:**
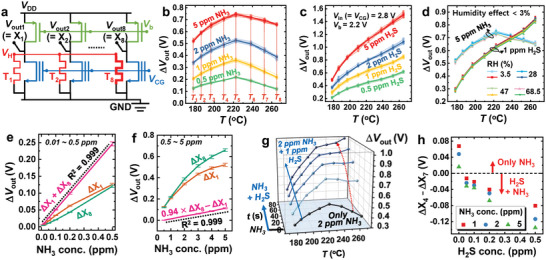
Characteristics of the artificial olfactory sensing unit array and its application. a) Schematic circuit diagram of the artificial olfactory sensing unit array. b) Output signals (Δ*V*_out_s) of the array (ΔX_1_ − ΔX_8_) after the NH_3_ and c) H_2_S gas reactions. Δ*V*_out_ is the change in *V*_out_ before and after the gas reaction. d) Δ*V*_out_s of the array at various relative humidity (RH). e) LCs of Δ*V*_out_s. ΔX_1_ + ΔX_8_ and f) 0.94 × ΔX_8_−ΔX_1_ are linearly related (*R* ^2^ = 0.999) to the NH_3_ gas concentration in the 0.01–0.5 ppm and 0.5–5 ppm concentration ranges, respectively. g) Δ*V*_out_s of the array when 1 ppm H_2_S gas is generated in the presence of 2 ppm NH_3_ gas. h) ΔX_4_−ΔX_7_ in various NH_3_ and H_2_S gas mixture environments.

The artificial olfactory sensing unit array consists of eight sensing units with different operating temperatures (*T*_1_–*T*_8_) and output signals (X_1_–X_8_) (**Figure** **4**a). When the temperatures (*T*_1_–*T*_8_)of eight microheaters are 178–265 °C (*V*_H_ = 3 V and *R*_H_ = ∼400 Ω), the power consumption of the array is 22.6 mW (Figure S7, Supporting Information). Due to the excellent thermal insulation structure of the microheaters,^[^ ^45^ ^]^ the total power consumption levels of eight sensing units are comparable to those of typical commercial single gas sensors (15–60 mW).^[^ ^61^ ^]^ Figure 4b,c exhibit the Δ*V*_out_s of the array response to NH_3_ and H_2_S gas, respectively. The Δ*V*_out_s for NH_3_ and H_2_S gases have different patterns. NH_3_ gas has a convex upward Δ*V*_out_ pattern, whereas H_2_S gas has a pattern in which Δ*V*_out_ increases as *T* increases due to the sulfuration reaction.^[^ ^59^ ^]^ The array is tested in humidity‐changing environments similar to those of real applications; there is little (<3%) decrease in Δ*V*_out_s with increasing relative humidity (RH) (Figure 4d; Tables S2 and S3, Supporting Information). The *V*_out_ baseline change of sensing units with varying humidity is shown in Figure S8 (Supporting Information).

When the meat starts to rot, NH_3_ gas is generated, the decay worsens, the NH_3_ concentration gradually increases, and H_2_S gas is produced. Therefore, monitoring the concentration of NH_3_ gas and the presence of H_2_S gas is essential for meat spoilage detection. The NH_3_ and H_2_S gas concentration versus output signal curves have nonlinearities in all eight sensing units and follow the Langmuir adsorption model (Figure S9, Supporting Information).^[^ ^62^ ^]^ Due to this inherent nonlinearity, in previous studies, the gas concentration is predicted by sending the output signals to the server and applying them to nonlinear functions.^[^ ^7^, ^63^ ^]^ Herein, the proposed AOS uses linear combinations (LCs) of the sensing unit outputs that have linear relationships with the NH_3_ gas concentration. Two LCs (ΔX_1_+ΔX_8_ and 0.94 × ΔX_8_ −ΔX_1_) are used to generate signals that are linear over the 0.01–0.5 ppm and 0.5–5 ppm NH_3_ gas concentration ranges, respectively (Figure 4e,f). The latter LC is intentionally set to have a negative value when the NH_3_ gas concentration is <0.5 ppm; thus, the LC is only utilized when its value is positive. Notably, it is feasible to generate LCs with linearity in gas concentrations across various concentration ranges. In other words, the LC configuration can be tailored to the purpose and widely applied. LCs can be used as binary information (positive or negative) and analog numeric values. For example, after setting two LCs, food conditions can be considered fresh if both LC values are negative, edible if the former LC value is positive and the latter LC value is negative, and spoiled if both LC values are positive. The proposed AOS can detect the presence of H_2_S gas. Since NH_3_ and H_2_S have distinct Δ*V*_out_ patterns, the pattern is significantly altered when H_2_S is generated in an environment containing NH_3_ gas (Figure 4g). The generation of H_2_S gas dramatically increases the Δ*V*_out_ values of sensing units operating at high temperatures. The existence of H_2_S gas can be confirmed by simply comparing X_4_ and X_7_. ΔX_7_ is greater than ΔX_4_ in environments containing H_2_S gas. (Figure 4f).

### 2.3 Characteristics of the Artificial Olfactory Computing Unit

**Figure 5:**
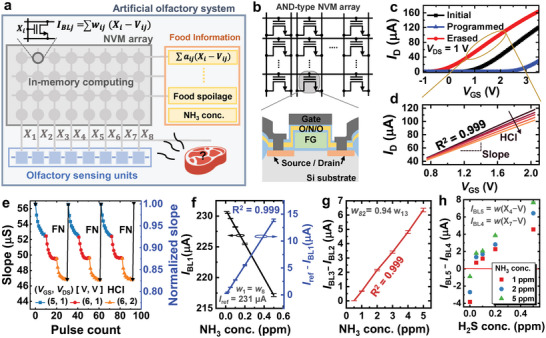
Characteristics of the artificial olfactory computing unit. a) Schematic diagram of the AOS and b) the computing unit (AND‐type NVM array). A schematic cross‐section of an NVM cell is shown. c) Program and erase characteristics of the NVM cell. d) Slope of the transfer curve (= transconductance) reduction by HCI. The graph shows an enlarged view of the part of the transfer curves where *V*_GS_ and *I*_D_ show excellent linearity (*R* ^2^ = 0.999). e) Slope change in the NVM cell with the HCI pulse. Blue squares, red circles, and yellow triangles indicate when HCI is performed for 100 µs under (*V*_GS_, *V*_DS_) = (5, 1 V), (6, 1 V), and (6, 2 V) conditions, respectively. The slope changed by HCI returns to the initial state by successively performing two FN tunneling steps ((*V*_GS_, *t*_pulse_) = (−9 V, 100 µs) + (10 V, 100 µs)). f). *I*_BL1_ (= *w*(X_1_+X_8_ − 2 *V*)) and *I*_ref_−*I*_BL1_ versus NH_3_ gas concentration. g) *I*_BL3_−*I*_BL2_ (= *w*(X_1_ − 0.94  ×  X_8_)) versus NH_3_ gas concentration. h) *I*_BL5_−*I*_BL4_ (= *w*(X_4_ − X_7_) in various NH_3_ and H_2_S gas mixture environments.

The artificial olfactory computing unit consists of the AND‐type NVM array. The output signals of the sensing units are connected to the WLs of the NVM array. The signals are linearly combined through IMC‐based MAC operations and output as BL currents (*I*_BL_s) (**Figure** **5**a). The *I*_BL_ can be expressed as follows:

(1) $$I_{\mathrm{BLj}}=\sum_{i}w_{\mathrm{ij}}\left(X_{i}-V_{\mathrm{ij}}\right)$$

where *w*_ij_ and *V*_ij_ are the transfer curve slope and the *V*_th_ of the NVM cell, respectively. The AND‐type NVM array is utilized due to its high density and programmability with low power using Fowler–Nordheim (FN) tunneling (Figure 5b).^[^ ^64^ ^]^ The NVM cells can be programmed/erased using a poly‐Si floating gate as a charge storage layer (Figure 5c). Unnecessary NVM cells are programmed and turned off so that specific X_i_s contribute to the *I*_BL_, and the cells are programmed/erased to have the desired *V*_ij_. The slopes (*w*_ij_s) of NVM cells can be controlled by adjusting the interface trap density using hot carrier injection (HCI) (Figure 5d).^[^ ^65^ ^]^ Figure 5e exhibits the slope change in the NVM cell when HCI is repeatedly performed for 100 µs while adjusting *V*_GS_ and *V*_DS_. The slope that is changed by HCI can be returned to the initial state by consecutively performing two FN tunneling steps (one erasing step (−9 V, 100 µs) and one programming step (10 V, 100 µs) at *V*_D_ = *V*_S_ = 0 V) (Figure S10, Supporting Information). Since the NVM cells show excellent retention characteristics at 300 K (Figure S11, Supporting Information), an AOS with uniform performance can be obtained with a single configuration.

The proposed IMC method effectively provides LCs of signals. If two of the NVM cells connected to X_1_ and X_8_ are turned on with the same slope and the other NVM cells are turned off, *I*_BL1_ with a linear relationship to the NH_3_ gas concentration (0.01–0.5 ppm range) can be obtained (Figure 5f). By using an appropriate *I*_ref_, an *I*_ref_−*I*_BL1_ linear relationship to the NH_3_ gas concentration can be obtained (Figure 5f). Notably, the difference between the two currents can be obtained with a simple circuit.^[^ ^66^ ^]^ Similarly, LC with linearity in high‐concentration NH_3_ gas (Figure 5g) and LC indicating the presence of H_2_S gas (Figure 5h) can be successfully implemented using the NVM array. The proposed AOS can output various LCs that provide meaningful olfactory information by repeatedly using sensing signals. Note that the proposed low‐energy processing method is an efficient alternative to data processing and inference performed on conventional external processors and servers.

### 2.4 Application to Meat Spoilage Detection

**Figure 6:**
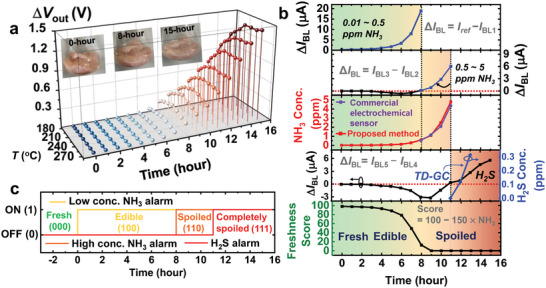
Meat spoilage tracking using the proposed AOS. a) Transient Δ*V*_out_s during the spoilage of chicken tenderloin. The images of the food are shown in the insets. b) Δ*I*_BL_s, system‐inferred NH_3_ gas concentration, commercial electrochemical sensor‐inferred NH_3_ gas concentration, TD‐GC‐inferred H_2_S gas concentration, system‐inferred H_2_S gas generation time, and freshness score during food spoilage. The NH_3_ gas concentrations in the ranges of 0.01–0.5 ppm and 0.5–5 ppm are inferred using *I*_ref_−*I*_BL1_ and *I*_BL3_−*I*_BL2,_ respectively. The generation of H_2_S gas is inferred using the change in the sign of *I*_BL5_−*I*_BL4_ from negative to positive. The freshness score is calculated using Score = 100−150 ×  NH_3_ gas concentration. If the food generates >0.5 ppm of NH_3_ gas, a freshness score of 0 is assigned. c) Method of classifying the food condition using the three outputs of the AOS as binary signals. The system provides binary signals whether 1) the NH_3_ gas concentration is >0.01 ppm (low conc. NH_3_ alarm, yellow line), 2) the NH_3_ gas concentration is >0.5 ppm (High conc. NH_3_ alarm, orange line), and 3) H_2_S gas is generated (H_2_S alarm, red line). By using three binary signals, the food conditions are labeled fresh (000), edible (100), spoiled (110), and completely spoiled (111).

The meat spoilage detection ability of the proposed AOS is tested using chicken tenderloin. The Δ*V*_out_s of the sensing units and images of the food over time are shown in **Figure** **6**a. Chicken tenderloin is kept at room temperature for testing. As the meat spoils, it releases various gases including NH_3_ and H_2_S. On the assumption that NH_3_ and H_2_S gases, which are predominantly produced during meat spoilage, have a dominant influence on the sensor signal, NH_3_ gas concentration prediction and H_2_S gas generation detection are carried out. The reference point (0 h) is chosen when the amount of NH_3_ gas produced from food exceeds 0.01 ppm. As food spoils, NH_3_ gas is generated, its concentration increases, and Δ*V*_out_s increases. Afterward, the Δ*V*_out_ pattern changes as H_2_S gas is generated. The outputs of the sensing units are processed in the integrated NVM array to identify NH_3_ gas concentration and food condition (Figure 6b). The NH_3_ gas concentration surpasses 0.5 ppm after eight hours. The *I*_BL3_–*I*_BL2_ that is negative becomes a positive value after 8 h. After 11 h, the concentration of NH_3_ gas exceeds 5 ppm, and H_2_S gas starts to be generated. The *I*_BL5_–*I*_B4_ changes from negative to positive after 11 h and continues to increase thereafter as the amount of H_2_S gas produced by food spoilage increases. This finding indicates that the food is completely spoiled from that point onward. To confirm that the proposed AOS accurately detects gases, the gases were analyzed using the commercial gas detection system using electrochemical sensors and gas chromatography using thermal desorption (TD‐GC) (Figure 6b). The concentration of NH_3_ gas predicted using the commercialized electrochemical sensor is similar to that predicted by the proposed AOS. The concentrations of H_2_S gas predicted by TD‐GC are 0.015 ppm and 0.118 ppm at 11 and 12 h, respectively. The generation time of a significant amount (>0.1 ppm) of H_2_S gas predicted using TD‐GC is 11 to 12 h, showing a similar trend to the proposed AOS.

The proposed AOS provides continuous and real‐time analog information related to food spoilage, and it can provide appropriate food freshness scores for target foods. Herein, as an example, we set the freshness score to 100−150 ×  NH_3_ gas concentration (Figure 6b). We set the freshness score to zero (0) if the food produces >0.5 ppm of NH_3_ gas. The three output signals of AOS can be used as binary digital alarms to provide food condition information. They change from negative (state 0) to positive (state 1) in the presence of low‐concentration NH_3_ (0.01 ppm), high‐concentration NH_3_ (0.5 ppm), and H_2_S gas, respectively (Figure 6c). By using the three binary signals, the state of food can be divided into fresh (000), edible (100), spoiled (110), and completely spoiled (111). Unlike visual inspection, which can only determine food conditions when it is completely spoiled, the proposed system can track and evaluate the entire food spoilage process.

## 3 Conclusion

In summary, we have proposed an artificial olfactory system for application to food spoilage detection. The proposed AOS efficiently integrates sensing and computing units on the same chip, and it provides meaningful information by processing raw data through near‐sensor computing. The sensing units have high sensitivities to NH_3_ and H_2_S gases, which are essential for detecting food spoilage and insensitive to humidity. The sensing units exhibit very low detection limits (<0.01 ppm NH_3_ and 0.01 ppm H_2_S) because of the thin‐film ZnO gas sensing material that considers the sensor transducer, high amplifier gain, and low noise. The eight sensing units configured in the array operate at different temperatures, resulting in different gas sensing characteristics; the signals from the sensing units show different patterns for NH_3_ and H_2_S gases. Linear combinations of sensing unit signals that are linear to NH_3_ gas concentration and notify H_2_S gas generation are calculated through in‐memory computing (IMC) in the integrated non‐volatile memory (NVM) array. Proof‐of‐concept measurements of food spoilage are performed using chicken tenderloins. The proposed artificial olfactory system (AOS) can continuously monitor food throughout the spoilage process, estimate the NH_3_ gas concentration and freshness score based on analog outputs, and classify food conditions using binary signals.

Here, AOS is designed considering NH_3_ and H_2_S gases, which are the most representative markers of meat spoilage. Since the sensor is primarily influenced by the two gases produced by meat, the predicted NH_3_ gas concentration and H_2_S gas generation time are comparable to those obtained with commercial gas detection analysis equipment (electrochemical sensor and TD‐GC). The proposed AOS judged food spoilage conditions based on the detection of these two gases. However, to determine the degree of food spoilage in detail, various volatile organic compounds (VOCs) should be considered. We believe that the proposed AOS will achieve better performance in food spoilage detection if sensing characteristics of sensors to various VOCs are considered.

Unlike conventional systems, the proposed AOS has a high energy efficiency and compact design because it does not need to convert all raw data using ADCs and perform low‐level processing. The proposed AOS processes the analog sensing signal without interface circuits (e.g., ADCs) between the sensors and the processor and converts it into meaningful refined data on the same substrate integrated with the gas sensors. Since the AOS performs processing and computing at the edge, converting and transmitting only the minimum essential data, it minimizes energy consumption and latency. The AOS is believed to have significant potential for edge device applications requiring ultralow energy. In this study, the proposed AOS is specialized for food spoilage detection; however, the proposed AOS platform can be used for various applications by adjusting the gas‐sensing materials and operating temperature ranges and by programming the NVM cells.

## 4 Experimental Section

### Device Fabrication

The artificial olfactory system was fabricated in a cleanroom at the Inter‐university Semiconductor Research Center (Seoul National University) by utilizing conventional CMOS processes with ten photomasks on a 6‐inch *p*‐type bulk Si wafer with an orientation of (100) (Figure S1, Supporting Information). The wafers were cleaned using a sulfuric acid peroxide mixture (SPM), ammonia peroxide mixture (APM), hydrochloric acid and peroxide mixture (HPM), and diluted hydrofluoric acid (DHF) solutions. Reference markers were patterned using inductively coupled plasma (ICP) dry etching on Si substrates. A 10‐nm‐thick SiO_2_ layer was deposited by low‐pressure chemical vapor deposition (LPCVD) to create a sacrificial oxide that could protect the substrate throughout the ion implantation process. For the *p*‐channel devices, an *n*‐well implantation procedure (P^+^, 120 keV, 3 × 10^12^ cm^−2^) was performed. The *n*‐well with a depth of ∼2 µm was produced by performing the drive‐in operation for 11 h at 1100 °C. Following the removal of the sacrificial oxide, a 10‐nm‐thick SiO_2_ layer, and a 150‐nm‐thick Si_3_N_4_ layer were successively produced on the substrate by thermal oxidation and LPCVD, respectively. The two layers were successively patterned by the reactive ion etching (RIE) method to define the active areas of the FETs. Then, B^+^ field implantation (40 keV, 1.6 × 10^13^ cm^−2^) was conducted. By using the local oxidation of silicon (LOCOS) technology, a 550‐nm‐thick SiO_2_ layer (field oxide) was thermally grown to electrically isolate nearby devices. The SiO_2_ and Si_3_N_4_ layers above the active areas were removed using wet etching with hot phosphoric acid (H_3_PO_4_) and DHF solutions. As the final step of the LOCOS technique, the thermal oxidation process was conducted to remove the white ribbon‐shaped residues created during the wet oxidation process. After ion implantation to adjust the threshold voltage (*V*_th_), the thermal oxide film was removed from the DHF solution. A 10‐nm‐thick SiO_2_ layer (gate oxide) was thermally grown. A 300‐nm‐thick in situ *n* ^+^‐doped poly‐Si was produced by LPCVD and patterned for floating gates for NVMs and sensors, gates for FET loads, and microheaters for sensors. Source/drain (S/D) implantation (*n*‐channel: As^+^, 40 keV, 2 × 10^15^ cm^−2^, *p*‐channel: BF_2_^+^, 25 keV, 2 × 10^15^ cm^−2^) was conducted. The O/N/O passivation layer (10/20/10 nm) was successively deposited following the rapid thermal process (RTP) (1050 °C, 5 s). After defining the contact holes, Ti/TiN/Al/TiN metal layers (20/20/50/10 nm) were deposited and patterned for control gates for sensors and NVMs and metal electrodes. The H_2_ alloying process was conducted at 400 °C for 10 min. Etching holes were patterned, and two kinds of dry etching were conducted to create an air gap underneath the heater. To etch the field oxide, ICP etching using CF_4_ gas was performed. Then, an RIE method using SF_6_/Ar gas was used to etch the Si substrate isotropically. Finally, the sensing material (ZnO) was deposited and patterned.

### ZnO Characterization

Surface scanning electron microscopy (SEM) and atomic force microscopy (AFM) images of the ZnO film were obtained using a field‐emission scanning electron microscope (SIGMA, Carl Zeiss) and atomic force microscope (NX‐10, Park Systems). The GIXRD pattern was obtained using a grazing incidence X‐ray diffractometer (Xpert Pro, PANalytical). The XPS data were obtained using the VersaProbe III scanning XPS microprobe equipped with an Al Kα source.

### Gas‐Sensing Measurement

Measurements of sensor, including food spoilage detection, were conducted using a probe station (main chamber) with gas injection and ejection and a semiconductor parameter analyzer (B1500A, Agilent) (Figure S12, Supporting Information). Humidity and gas concentration levels were adjusted in the mixing chamber using the mass flow controller (MFC), and gas was injected into the main chamber at a rate of 200 mL min^−1^. Dry air was bubbled through deionized water to form humid air. The mixing ratio of humid air and dry air was adjusted to form humid air with the desired relative humidity. Afterward, a humidity test was performed using a commercial humidity sensor. For food spoilage detection, dry air was injected into the container with the food sample at a rate of 200 mL min^−1^, and gases from the food were injected into the main chamber. Gases from the food sample (chicken tenderloin) were analyzed using a commercial gas detection system using electrochemical gas sensors (AOMS‐1000, ACEN) and gas chromatography with thermal desorption (TD‐GC, TurboMatrix 300 TD & Clarus 690 GC, PerkinElmer). The gases were contained in Tedlar bags and used for analysis. The gases generated from the food at approximately the same time were utilized for the proposed AOS and commercialized systems.

The simplified equivalent circuit and *I*_H_–*V*_H_ curve of the microheater array are shown in Figures S13a and S7a (Supporting Information). The temperatures of the sensing units belonging to the array were predicted through a comparison of output signals of a single sensing unit and of the array unit (Figure S13b, Supporting Information). Figure S7 (Supporting Information) shows the temperature and power characteristics of the microheaters. A single sensing unit was relatively free from parasitic resistance characteristics, such as line resistance, making temperature predictable using conventional resistance temperature detection (RTD) methods.^[^ ^34^ ^]^

## Conflict of Interest

The authors declare no conflict of interest.

## Supporting information

Supporting Information

## Data Availability

The data that support the findings of this study are available from the corresponding author upon reasonable request.
