# Supplementary material for: Energy Efficient Artificial Olfactory System with Integrated Sensing and Computing Capabilities for Food Spoilage Detection
Source: Adv Sci (Weinh). 2023 Aug 31;10(30):2302506. doi: 10.1002/advs.202302506 (PMC10602532; doi:10.1002/advs.202302506)
Supplement: Supplementary file 1 — Supporting Information [file ADVS-10-2302506-s001.pdf]

ADVANCED SCIENCE
Open Access

Supporting Information

for *Adv. Sci.*, DOI 10.1002/advs.202302506

Energy Efficient Artificial Olfactory System with Integrated Sensing and Computing Capabilities for Food Spoilage Detection

*Gyuweon Jung, Jaehyeon Kim, Seongbin Hong, Hunhee Shin, Yujeong Jeong, Wonjun Shin, Dongseok Kwon, Woo Young Choi and Jong-Ho Lee**

Supporting Information

Energy Efficient Artificial Olfactory System with Integrated Sensing and Computing Capabilities for Food Spoilage Detection

*Gyuweon Jung, Jaehyeon Kim, Seongbin Hong, Hunhee Shin, Yujeong Jeong, Wonjun Shin, Dongseok Kwon, Woo Young Choi, and Jong-Ho Lee**

This file contains:

Supplementary Information Figure S1-S13, Table S1-3

References

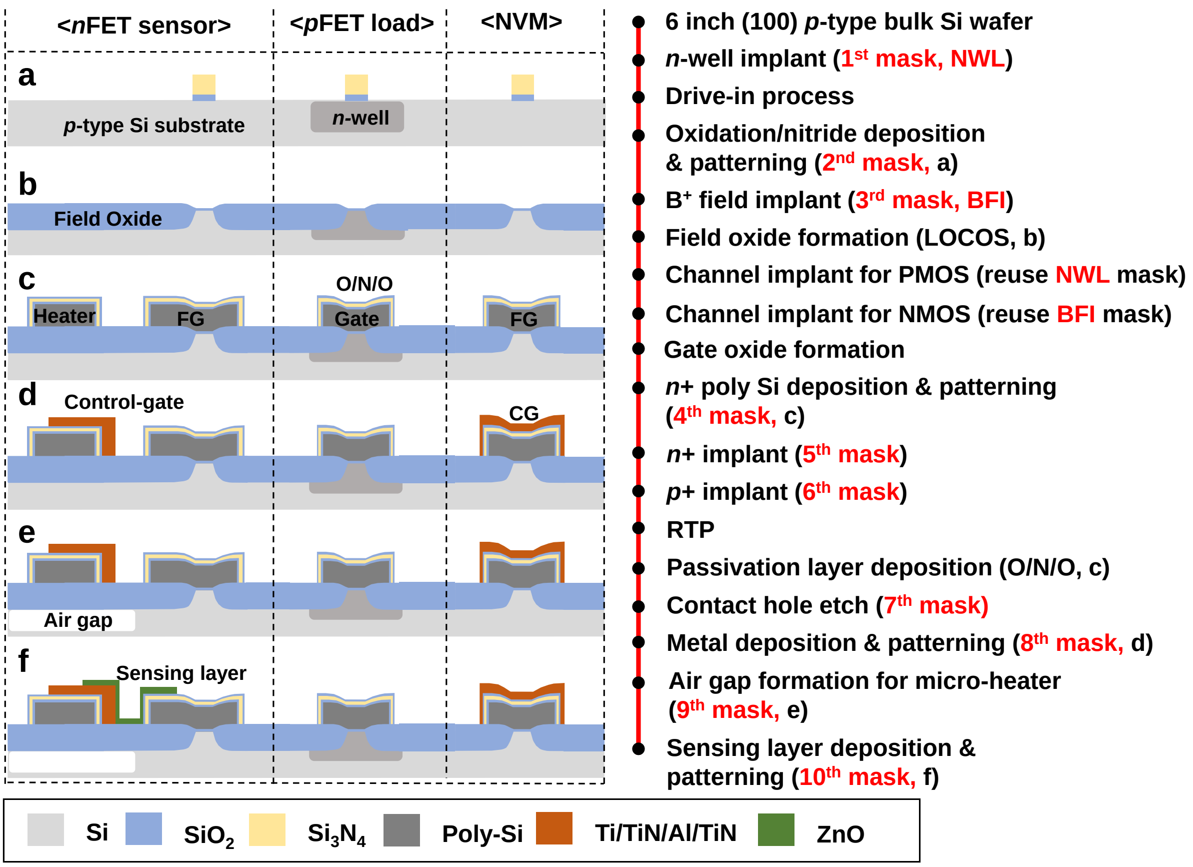

Figure S1. Key fabrication processes of the artificial olfactory system.

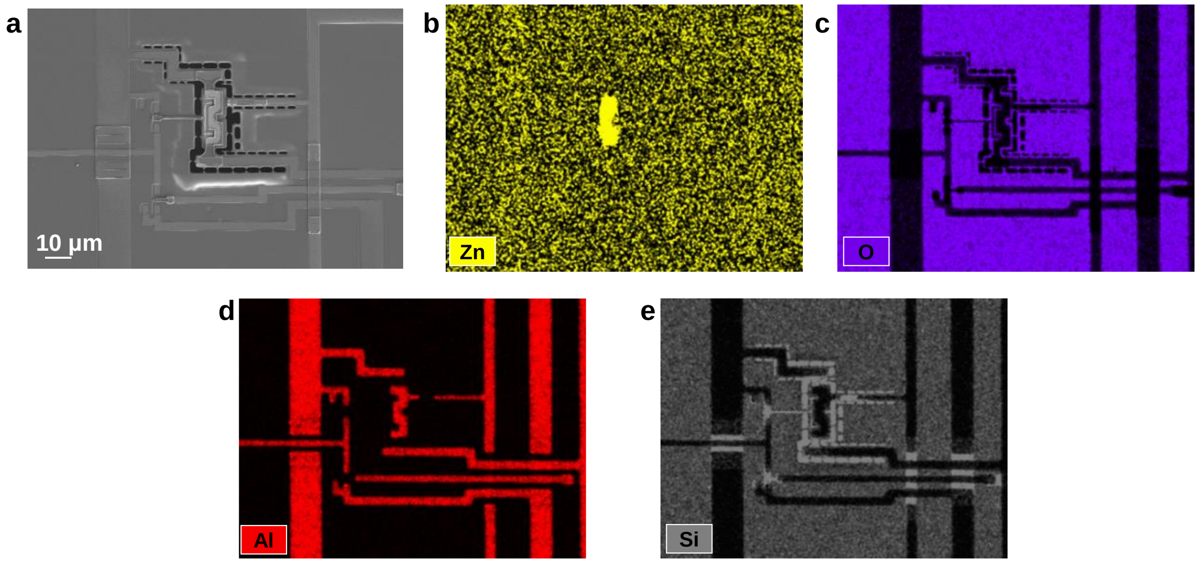

Figure S2. SEM images and EDS mapping images of the artificial olfactory sensing unit. a) Top SEM image of the fabricated artificial olfactory sensing unit. b–e) EDS mapping images of zinc (b), oxygen (c), aluminum (d), and silicon (e).

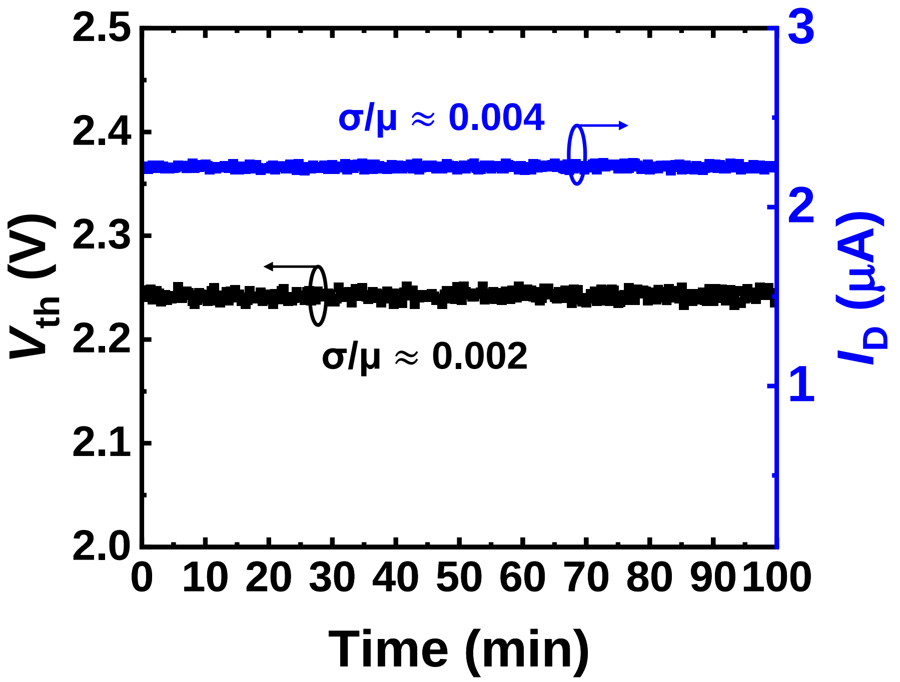

Figure S3. V_{th} and I_D over time for a FET-type sensor with applied operating voltages (V_{DS} = 1 V, V_{GS} = 2.8 V).

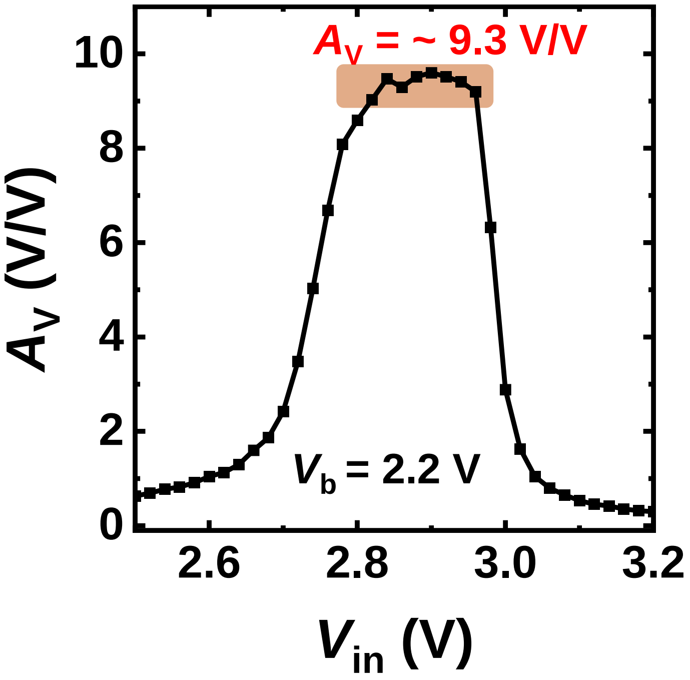

Figure S4. Gain of amplifier circuit as a function of input voltage (V_{in}) of FET-type gas sensor. The voltage (V_b) applied to the gate of the *p*FET load is 2.2 V.

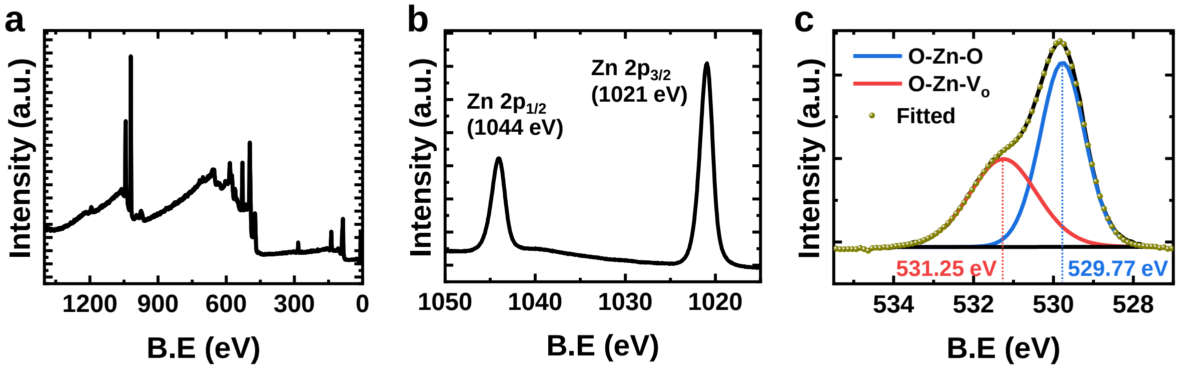

Figure S5. XPS spectra of the ZnO film. a–c) Wide-scan XPS spectrum (a) and high-resolution XPS spectra of Zn 2p (b) and O 1s (c).

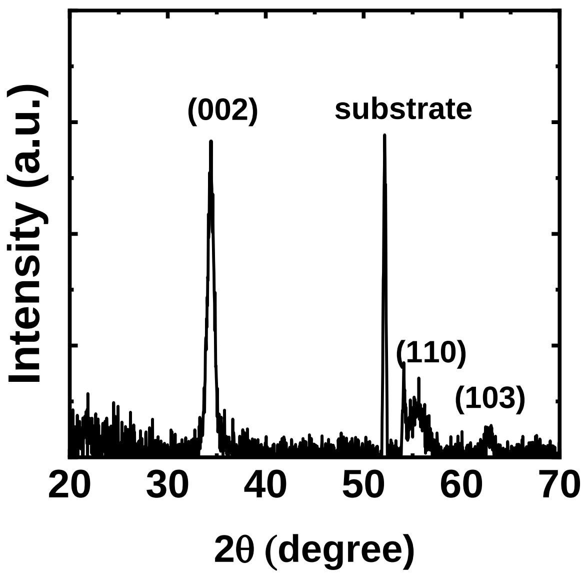

Figure S6. GIXRD of the ZnO film.

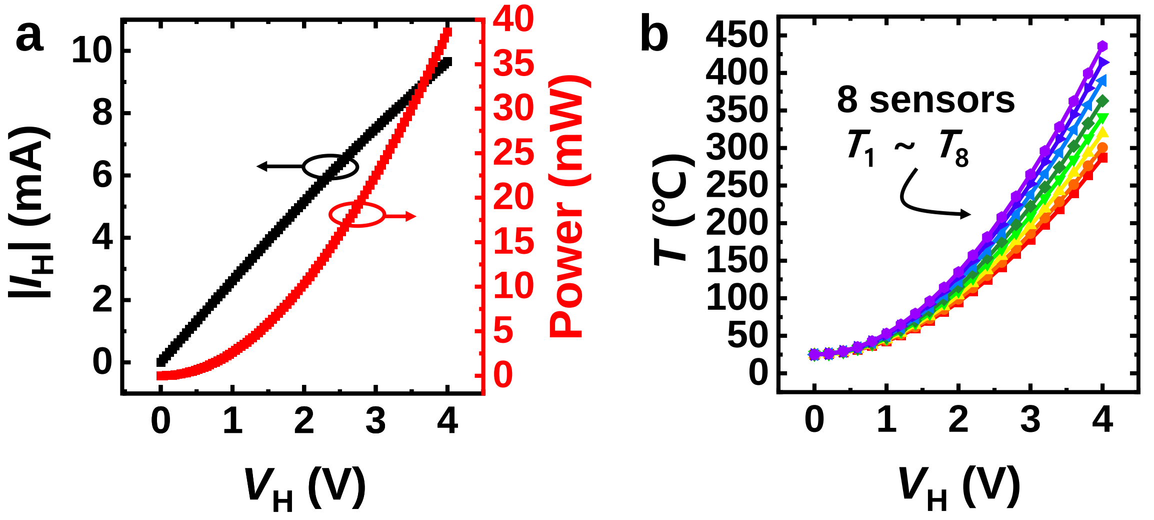

Figure S7. a) I_H and power consumption versus V_H. b) Temperatures of microheaters (T_1~T_8) versus V_H.

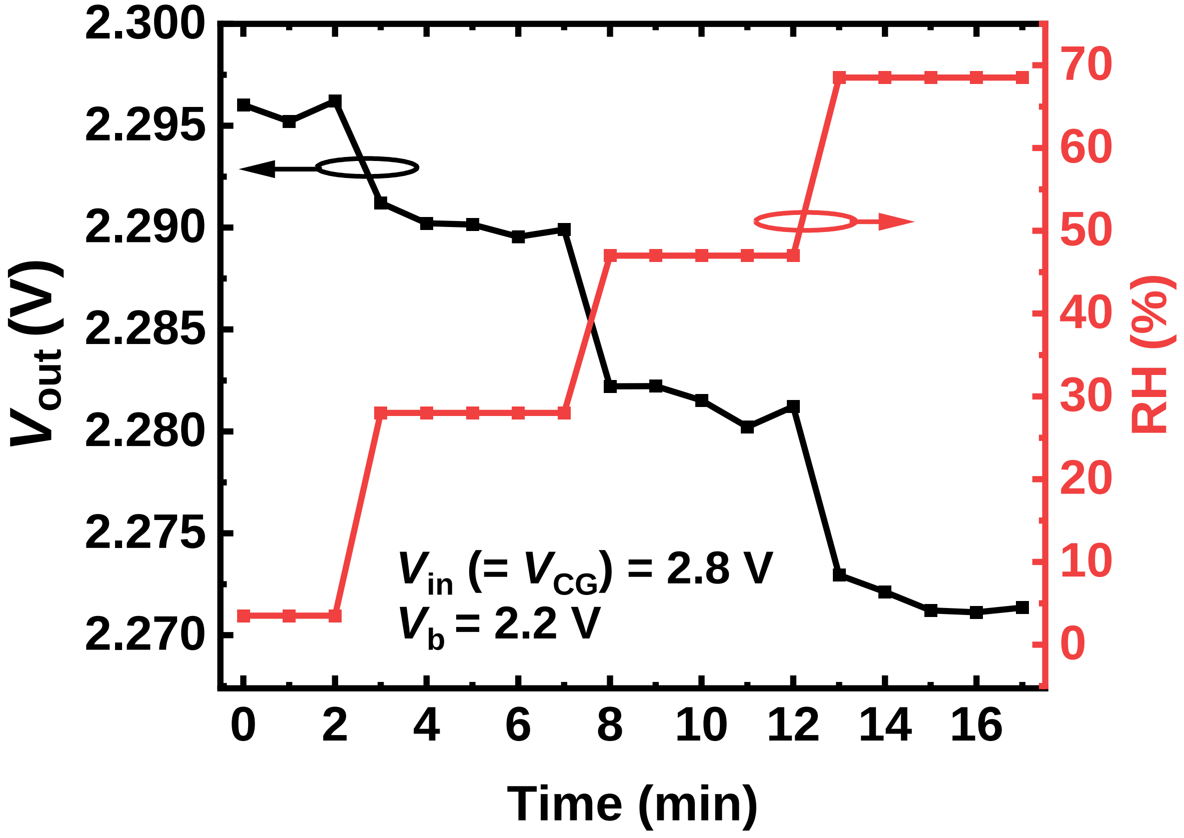

Figure S8. V_{out} baseline change with varying humidity

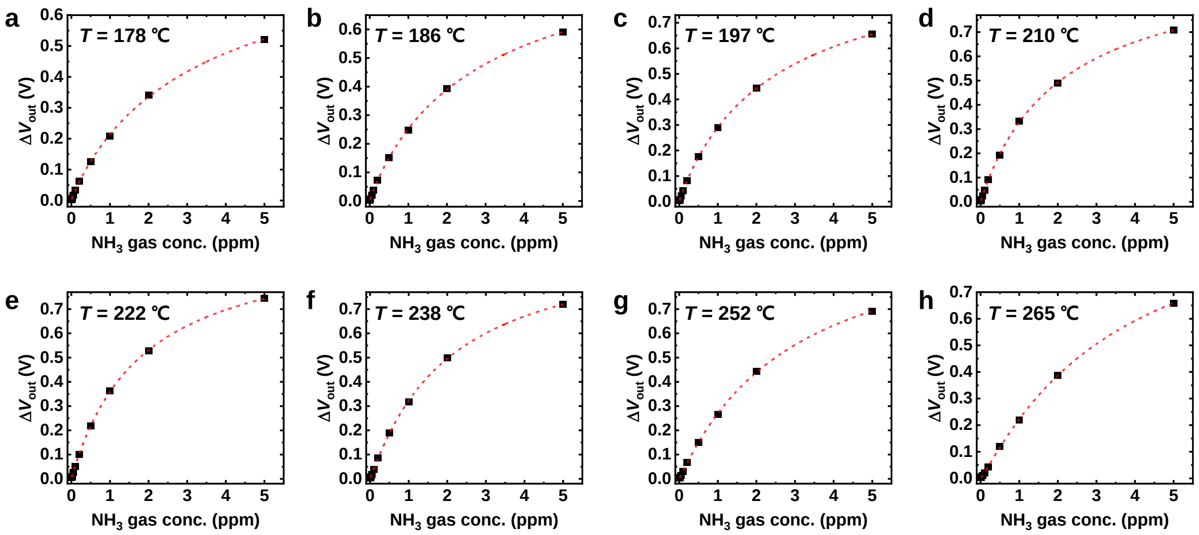

Figure S9. NH_3 and H_2S gas concentration versus output signal curves of sensing units. Each curve is well fitted to the Langmuir adsorption theory ($R^2 > 0.99$).

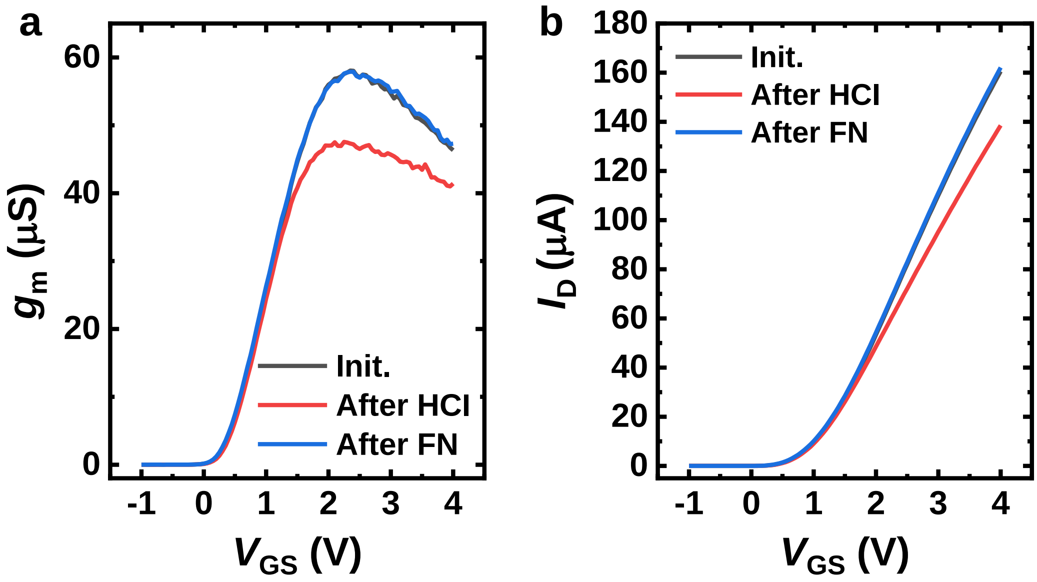

Figure S10. a–b) g_m (a) and I_D (b) versus V_{GS} curves of NVM cells in the initial state and after HCI operation and after additional FN tunneling operation. When FN tunneling is performed on an NVM cell that has undergone HCI, the curves return to their initial state.

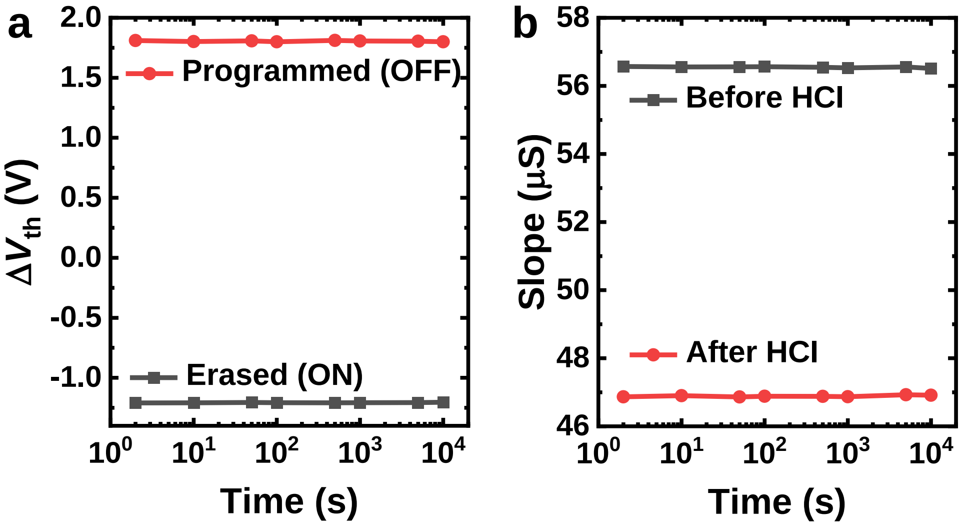

Figure S11. a–b) Retention characteristics for the ΔV_{th} (a) and slope (b) of the NVM cell at 300 K.

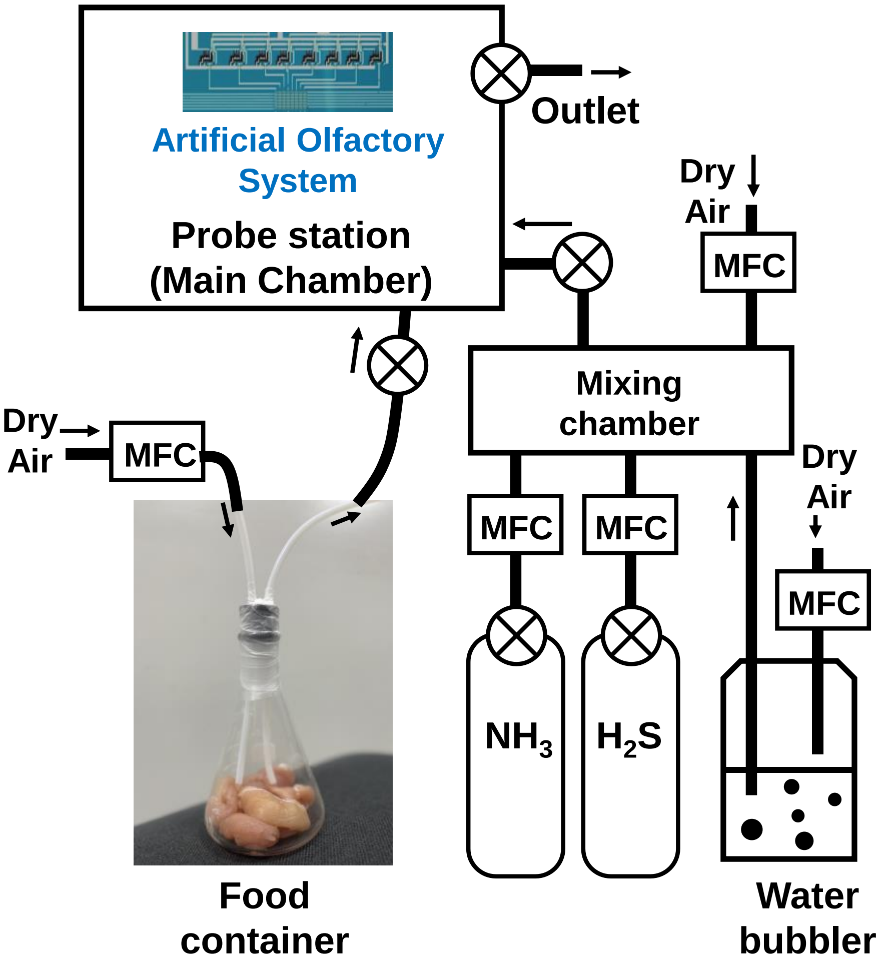

Figure S12. Schematic diagram of the gas measurement system.

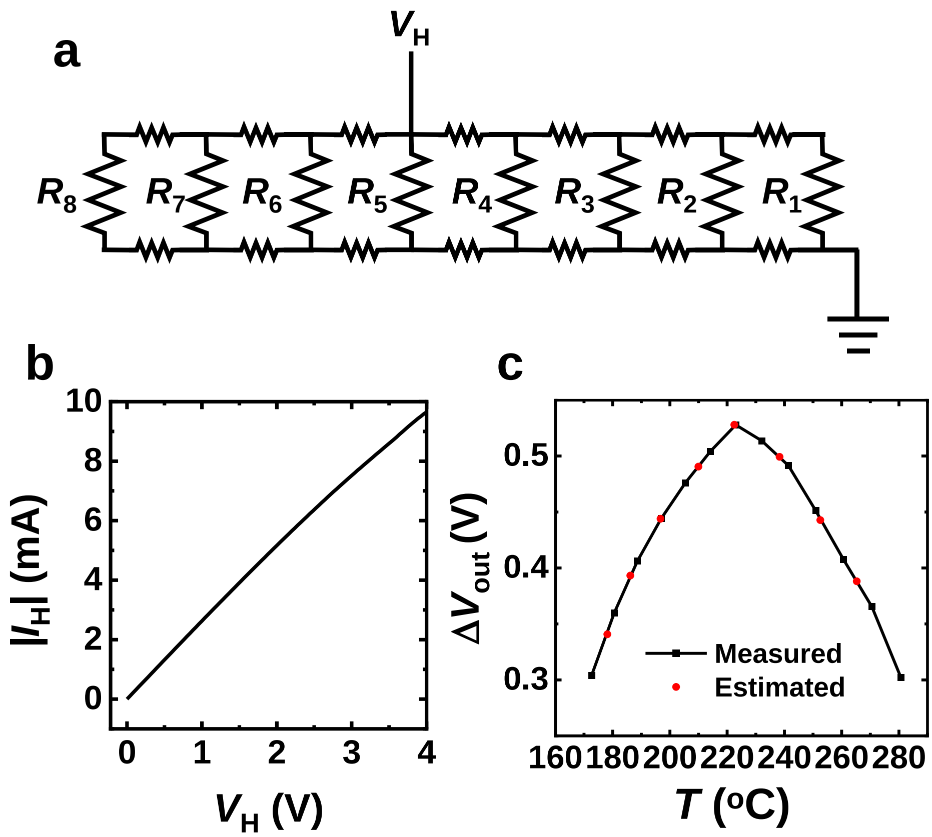

Figure S13. Method for measuring power consumption and temperature of microheater array. a) Schematic diagram of the microheater array. b) I_H–V_H curves of the microheater array. c) Temperature estimate of the sensing units in the array through comparison with the output signal of a single sensing unit.

Table S1. Performance comparison with state-of-the-art AOSs with sensing and computing capabilities.

Specifications	Application	Device size	Sensing material	Data processing method	Prediction method
**This work**	Food spoilage detection	~ 60 × 60 μm^2 (sensor + amp circuit, sensing unit), 6F^2 NVM cell	Simple ZnO film	Integrated circuit & NVM array (within the chip)	Addition/subtraction of sensor data
Nano Energy (2021)[S1]	Food spoilage detection	6 × 6 mm^2 (single sensor)	Sr-ZnO nanoparticles	RRAM (within) & Electrochemical actuators (separate chip)	On/off using threshold
Nature (2017)[S2]	Beverage classification	1.7 × 2.2 cm^2 (whole chip)	Carbon nanotube	RRAM, sense amplifier, multiplexer, classification accelerator (3D chip)	*in situ* classification
IEDM (2021)[S3]	Disease identification	6.52 mm^2 (whole chip)	Doped SnO_2 (SnO_2+ Al_2O_3+ PdO)	Processing in sensor (PIS, feature extraction), computing-in-memory (CIM) macro, digital chip (one package)	Convolutional Neural Network
Adv. Sci. (2022)[S4]	Beverage classification	~ 400 × 400 μm^2 (single sensor)	SnO_2, WO_3 films	1T neuron, synapse in PCB (2X2) (separate chips)	Binary classification
InfoMat (2021)[S5]	Gas classification	Commercialized gas sensor (Taguchi gas sensor)	Metal oxide semiconductor	Reservoir computing system (volatile memristor), neural network classifier (nonvolatile memristor) (separate chips)	Classification

Table S2. ΔV_{out}s of the sensing array for 5 ppm NH_3 at various humidities.

	**3.5 %**	**28 %**	**47 %**	**68.5 %**
**ΔV_{out1}**	0.5235	0.5192	0.5076	0.5128
**ΔV_{out2}**	0.5904	0.5829	0.5731	0.5768
**ΔV_{out3}**	0.6570	0.6513	0.6369	0.6387
**ΔV_{out4}**	0.7128	0.6985	0.6882	0.6938
**ΔV_{out5}**	0.7512	0.7415	0.7293	0.7329
**ΔV_{out6}**	0.7208	0.7134	0.6955	0.6882
**ΔV_{out7}**	0.6902	0.6815	0.6749	0.6731
**ΔV_{out8}**	0.6608	0.6511	0.6480	0.6452

Table S3. ΔV_{out}s of the sensing array for 1 ppm H_2S at various humidities.

	**3.5 %**	**28 %**	**47 %**	**68.5 %**
**ΔV_{out1}**	0.2945	0.2928	0.2909	0.2847
**ΔV_{out2}**	0.3924	0.3920	0.3888	0.3813
**ΔV_{out3}**	0.4871	0.4820	0.4787	0.4762
**ΔV_{out4}**	0.5654	0.5634	0.5577	0.5500
**ΔV_{out5}**	0.6373	0.6321	0.6261	0.6195
**ΔV_{out6}**	0.7017	0.6946	0.6906	0.6821
**ΔV_{out7}**	0.7631	0.7564	0.7515	0.7401
**ΔV_{out8}**	0.8496	0.8434	0.8338	0.8280

References

[S1] Z. Gao, S. Chen, R. Li, Z. Lou, W. Han, K. Jiang, F. Qu, G. Shen, *Nano Energy* 2021, *86*, DOI 10.1016/j.nanoen.2021.106078.

[S2] M. M. Shulaker, G. Hills, R. S. Park, R. T. Howe, K. Saraswat, H. S. P. Wong, S. Mitra, *Nature 2017 547:7661* 2017, *547*, 74.

[S3] Z. Li, S. H. Sie, J. L. Lee, Y. R. Chen, T. I. Chou, P. C. Wu, Y. T. Chuang, Y. Te Lin, I. C. Chen, C. C. Lu, Y. Z. Juang, S. W. Chiu, C. C. Hsieh, M. F. Chang, K. T. Tang, in *Technical Digest - International Electron Devices Meeting, IEDM*, 2021.

[S4] J. K. Han, M. Kang, J. Jeong, I. Cho, J. M. Yu, K. J. Yoon, I. Park, Y. K. Choi, *Advanced Science* 2022, *9*, DOI 10.1002/advs.202106017.

[S5] T. Wang, H. M. Huang, X. X. Wang, X. Guo, *InfoMat* 2021, *3*, DOI 10.1002/inf2.12196.
